# A systematic review and meta-analysis of the use of drug-coated balloon angioplasty for treatment of both de novo and in-stent coronary chronic total occlusions

**DOI:** 10.1007/s00392-025-02639-y

**Published:** 2025-04-10

**Authors:** Rajkumar Natarajan, Natasha Corballis, Ioannis Merinopoulos, Vasiliki Tsampasian, Vassilios S. Vassiliou, Simon C. Eccleshall

**Affiliations:** 1 Department of Cardiology, Norfolk and Norwich University Hospital, 2.06 Bob Champion Research and Education Building, Norwich Medical School, University of East Anglia, Norwich, NR4 7TJ UK; 2 Norwich Medical School, University of East Anglia, Norwich, UK

**Keywords:** Drug coated balloon, Chronic total occlusion, Drug eluting stent, Percutaneous coronary intervention, CTO PCI

## Abstract

**Background:**

Modern contemporary percutaneous coronary intervention (PCI) techniques with drug-eluting stents (DES) have high procedural success rates in chronic total occlusion (CTO) but with a high prevalence of repeat revascularization. The use of drug-coated balloons (DCBs) in CTO is an alternative treatment strategy. The evidence for DCBs in CTO is, therefore, of interest, and we provide a structured and comprehensive review of the evidence available in terms of the use of DCBs in CTO, including de novo and in-stent (IS) CTO lesions.

**Objectives:**

We conducted a systematic review and meta-analysis on the use of DCBs in the management of coronary CTO.

**Methods:**

Electronic databases (PubMed, Embase and Ovid) were systematically searched from inception to April 2024 for DCB CTO studies. A meta-analysis was undertaken using a random-effects inverse-variance method due to heterogeneity. The primary outcome is target lesion revascularization (TLR). Secondary outcomes are major adverse cardiac events (MACE) as a composite of target lesion revascularization (TLR), cardiac death (CD), and any myocardial infarction (MI) including procedural and non-procedural MI, target vessel revascularization (TVR), angiographic outcomes such as late lumen loss (LLL), binary restenosis, and reocclusion.

**Results:**

A total of 10 studies consisting of 1,695 patients were systematically reviewed. This showed that late luminal changes in terms of lumen gain and minimal lumen loss were consistently seen in CTO cohorts 7–12 months after DCB treatment. Five studies were included for meta-analysis with 1,474 patients. There were no significant differences in TLR between treatment strategies such as DCB, DES, and hybrid (DES + DCB) in both de novo and IS-CTO populations as follows: DCB vs DES [OR, 0.71; 95% CI 0.49–1.02], DCB vs DES in IS-CTO [OR, 0.78; 95% CI 0.45–1.34], DCB vs Hybrid [OR, 0.96; 95% CI 0.39–1.43], and hybrid vs DES [OR, 0.76; 95% CI 0.15–3.84]. Similar findings were seen with the MACE outcome. A sensitivity analysis showed no difference between the above-mentioned groups in terms of MI, CD, and TVR.

**Conclusion:**

The limited initial evidence on DCB in coronary CTO-PCI suggests a safe and effective alternative treatment strategy and suggests RCTs are, therefore, required.

**Graphical Abstract:**

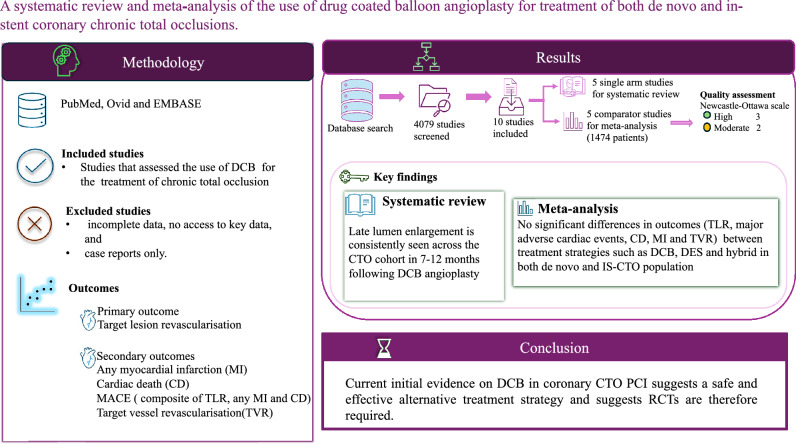

**Supplementary Information:**

The online version contains supplementary material available at 10.1007/s00392-025-02639-y.

## Introduction

A chronic total occlusion (CTO) is a complete occlusion of the coronary artery, with TIMI 0 flow through the lesion, but no evidence of thrombus, no staining at the proximal cap, and presence of mature collaterals with definitive corroborating evidence of occlusion duration ≥ 3 months as defined by the CTO-ARC consortium [1]. The prevalence of CTO varies from 15 to 20% of the patients undergoing coronary angiography [2–4] and higher incidence is found in patients with history of CABG [4]. CTO-PCI is a technically challenging procedure requiring additional skill sets and carries higher procedural risks [5, 6]. In the 2021 ACC/AHA [7] guidelines, CTO-PCI carries a class II-b/ level B evidence of recommendation, whereas in the 2019 ESC guidelines [8], CTO-PCI with a class II-a/ level B evidence is recommended for patients with refractory angina symptoms or with a large area of documented ischemia in the territory of the occluded vessel. The use of viability testing is heavily supported in the guidelines.

Technical and technological advances in coronary intervention have led to a much-improved success rate in CTO-PCI procedures in the past decade, dominated by the use of second- and third-generation DES and intravascular imaging techniques. Nevertheless, restenosis and stent failure (SF) remain high at 14–30% [5–7] in this unique subset of coronary lesions due to increasing lesion length, heavy calcification, lesion location (such as aorto-ostial or bifurcations), increased negative remodeling post-procedure, in-stent occlusions (IS-CTO), and stent factors including thickness, number, and design [8]. Furthermore, the adoption of aggressive algorithms to re-enter true lumen from the subintimal space predisposes to stent under-expansion and malapposition.

A meta-analysis comparing medical therapy and PCI in randomized studies for CTO showed no benefit in cardiac intervention [9]. It could be that the presence of the metallic stent limited the benefit from intervention for the reasons outlined above. Drug-coated balloons offer an alternative ‘no-metal’ local drug delivery strategy via a semi-compliant balloon technology [10] which could mitigate stent-related complications in CTO lesions. In de novo CTO lesions, DCB strategy may preserve coronary vasomotion, induce positive vessel remodeling, prevent stent-related complications, and reduce DAPT duration. While there are emerging evidence on use of DCBs in other subsets [11–13] of coronary lesions, including cost-effectiveness and mechanistic studies [14–16], the evidence on DCB in CTO remains scarce.

In this work, we sought to systemically review the available literature on use of DCB in coronary CTO lesions including de novo and IS-CTO.

## Methods

The study was performed based on the Preferred Reporting Items for Systematic Reviews and Meta-analysis (PRISMA) statement. Electronic databases, including PubMed, Embase, and Ovid, were comprehensively searched from inception until April 16th, 2024, using the MeSH terms “ (drug-coated balloon OR DCB OR DEB OR drug-eluting balloon) AND (chronic total occlusion OR CTO) AND (percutaneous coronary intervention)”. Clinical studies that assessed the use of DCB for the treatment of chronic total occlusion were included. Any study design was included. We excluded studies with incomplete data, no access to key data, and case reports only.

The primary outcome was target lesion revascularization (TLR). The secondary outcomes include major adverse cardiac events (MACEs) as a composite of target lesion revascularization (TLR), myocardial infarction(MI), cardiac death(CD). Other secondary outcomes were TVR, angiographic follow-up measures including late lumen loss, binary restenosis, late lumen gain, and reocclusion.

Two independent researchers (RN and NC) screened the abstracts individually, reviewed the full-text articles, and conflicts were resolved after discussion with a third researcher (VSV). Data were extracted from the included studies after full-text review and entered into a structured Excel spreadsheet comprising publication details, study design, baseline patient characteristics, procedural details, and outcomes. The study details that were extracted included: author, study design, year of publication, intervention, and sample size. The extracted baseline patient characteristics included age, sex, hypertension, diabetes, dyslipidemia, smoking, MI, previous PCI, prior CABG, clinical presentation, and LVEF. Procedural details that were extracted are as follows: access site, coronary artery intervened, J-CTO score (blunt stump, calcification, angulation, length > 20 mm and retry lesion), syntax score, DCB profile, DES profile, dissection types, and bailout stenting rates. Clinical outcomes that were available included: major adverse cardiovascular outcomes, TLR, MI, CD, target vessel revascularization, all-cause death, and angiographic outcome measures included reference vessel diameter, diameter stenosis %, late lumen loss, binary restenosis rate, reocclusion, and late lumen gain. The quality of the studies included for meta-analysis was assessed using the Newcastle–Ottawa scale [17]. Our study was registered with PROSPERO and the registration number is CRD42024569341.

## Statistical analysis

Statistical analysis was performed using the Review Manager software version 5.4 (The Nordic Cochrane Centre, Copenhagen, Denmark) on macOS software. Statistical heterogeneity was assessed by Chi-squared test (Cochrane Q) and *I*^2^ statistic test. In view of the differences in study designs, intervention arms, and outcome measures, a random-effects inverse-variance pooling model was used for all the meta-analyses independently of heterogeneity. Odds ratios (ORs) were reported with 95% confidence intervals (CIs). *P* < 0.05 was considered statistically significant. Sensitivity analysis was performed to obtain ORs for each of the MACE components, outcomes such as CD and MI.

## Results

### Study characteristics

**Fig. 1:**
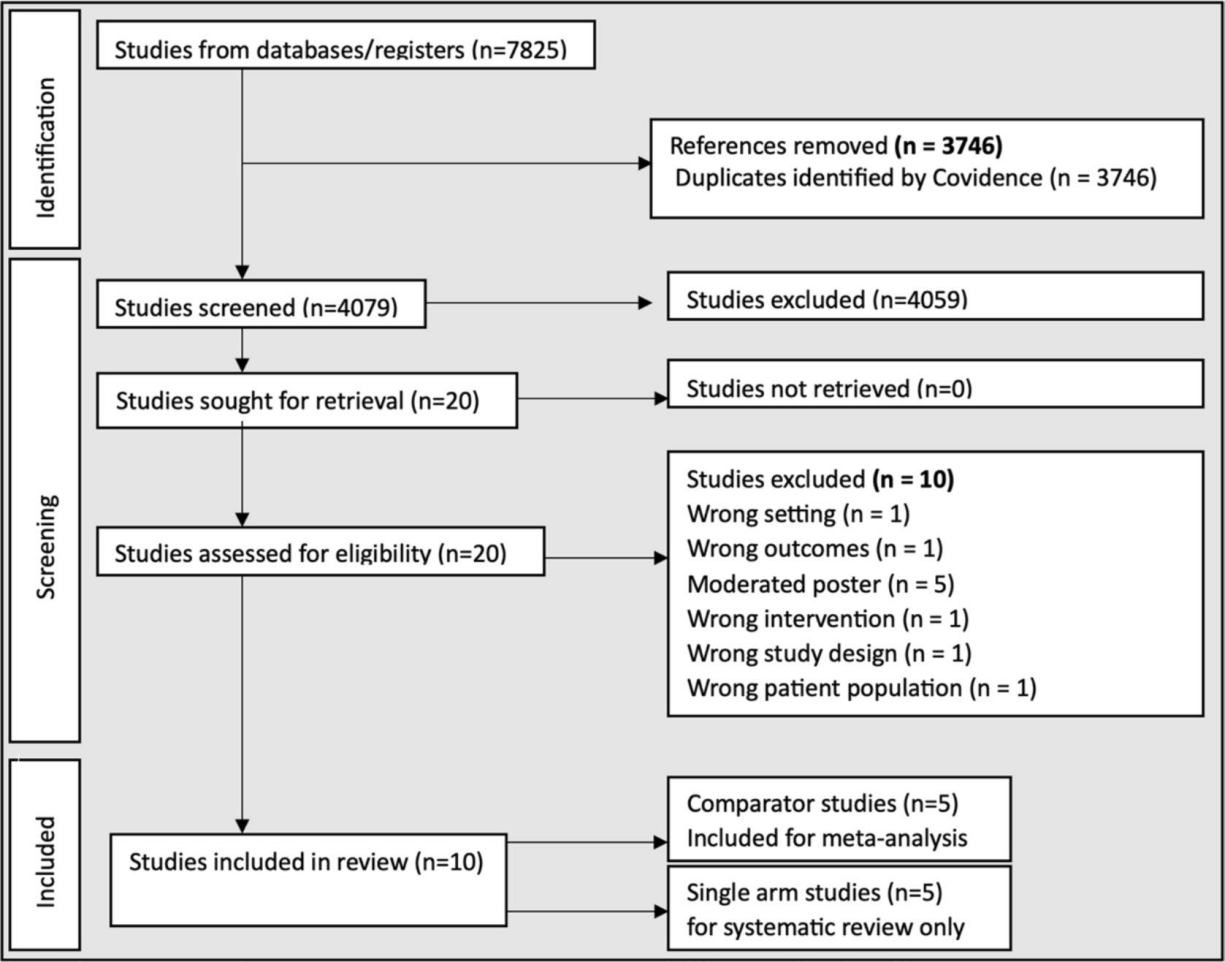
Search strategy

After screening 4079 studies, 10 clinical studies were identified for inclusion. For the five studies with a comparator arm [18–22], we have conducted a meta-analysis. The other five studies were single-arm studies [24–28] and have been discussed in a systematic review. Two of the five comparative studies exclusively compared DCB vs DES in in-stent-CTO population [21, 22]. For the studies included in the meta-analysis, there was significant methodological heterogeneity and as such, these have been grouped accordingly: (1) DCB vs DES, (2) DCB vs DES in IS-CTO, (3) DCB vs hybrid, and (4) hybrid vs DES. Figure 1 represents the PRISMA flowchart for study selection. Figure 1 represents the search strategy as per PRISMA guidelines.

The Newcastle–Ottawa scale (NOS) quality assessment of the five studies included for meta-analysis ranked 3 as high-quality studies and 2 as moderate quality, as shown in Table 1.

**Table 1:** Summaries the Newcastle–Ottawa scale for quality assessment

Study	Selection	Comparability	Outcome	Total	Quality
Basavarajaiah et al. [21]	***		***	6	Moderate
Zhang et al. [22]	***	***	***	8	High
Wang et al. [18]	****	**	**	8	High
Qin et al. [19]	****		***	6	Moderate
Madanchi et al. [20]	****	*	***	8	High

### Baseline clinical characteristics

Of the total 1695 patients from 10 studies, 65.7% were male with the mean age of 63.1 (IQR 58.3–69.8). Hypertension was highly prevalent (55.5%), followed by diabetes (34.6%). It is not uncommon for the studies to include a high number of CTO patients with prior PCI or CABG and was particularly notable in the studies with a hybrid (DCB + DES) arm. Table 2 below summarizes the study designs and baseline characteristics.

**Table 2:** Summary of study designs and baseline characteristics

First author/study/references	Year	Study design	Intervention (n)	Sample size	Age	Male n(%)	Smoking n(%)
Scheller et al. [23]	2016	Prospective feasibility study	DCB only (34)	34	59.18 ± 12.76	26(76.5)	5(14.7)
Onishi et al. [24]	2018	Prospective observational study	DCB only (12)	12	72 ± 6	5(45)	7(64)
Basavarajaiah et al. [21]	2021	Retrospective observational study	DCB(113) v DES(198) v POBA(88) in ISR CTO	403	69 ± 9.6	333(83.5)	153(38)
Onishi et al. [25]	2020	Retrospective observational study	DCB only (20)	20	72 ± 6	6(60)	6(60)
Jun et al. [26]	2022	Retrospective observational study	DCB only (84)	84	56.1 ± 9.9	72(85.7)	16(19.0)
Zhang et al. [22]	2022	Retrospective observational study	DCB (78) v DES (136) in IS-CTO	214	57.8 ± 9.0	179(83.6)	68(31.8)
Wang et al. [18]	2023	Prospective observational study	DCB (140) v hybrid (141) v DES(310)	591	58.4 ± 10.9	207(73.7)	110(39.9)
Qin et al. [19]	2023	Retrospective observational study	DCB (97) v hybrid (57)	154	60.2 ± 12.2	133 (86.4)	35 (22.7)
Terashita et al. [27]	2023	Retrospective observational study	DCB only (71)	71	67.7 ± 11.2	54(76.1)	23(32.4)
Madanchi et al. [20]	2024	Prospective observational study	DCB (46) vs hybrid (66) vs DES (43)	112	66 ± 10	100(89)	24(22)

First author/study/reference	DM *n* (%)	HTN *n* (%)	Dyslipidemia *n* (%)	Previous MI *n* (%)	Previous CABG *n* (%)	LVEF *n* (%)	Prior PCI *n* (%)
Scheller et al. [23]	8(23.5)	25(73.5)	19(55.9)	ND	ND	ND	ND
Onishi et al. [24]	6(55)	7(64)	6(55)	2(18)	ND	ND	4(36)
Basavarajaiah et al. [21]	201(50.4)	319(79.9)	ND	ND	24(6)	ND	404(100)
Onishi et al. [25]	4(40)	4(40)	6(60)	2(20)	ND	ND	5(50)
Jun et al. [26]	32(38.1)	49(58.3)	40(47.6)	21(25)	ND	50 ± 12.9	21(25)
Zhang et al. [22]	94(61.5)	134(62.6)	163(76.2)	113(52.8)	10(4.6)	62(58,66)	ND
Wang et al. [18]	105(37.4)	154(54.8)	150(53.4)	39(13.9)	4(1.4)	58.2 ± 7.0	39(13.9)
Qin et al. [19]	52(33.8)	100(64.9)	22(14.3)	43(27.9)	1(0.6)	59.4 ± 9.3	77(50)
Terashita et al. [27]	32(45.1)	57(80.3)	57(80.3)	26(36.6)	ND	55.7 ± 9.4	41(57.7)
Madanchi et al. [20]	38(35)	92(84)	88(79)	46(41)	11(10)	53 ± 10	ND

*DCB* drug-coated balloon, *DES* drug-eluting stent, *POBA* plain old balloon angioplasty, *IS-CTO* in-stent chronic total occlusion, *DM* diabetes mellitus, *HTN* hypertension, *LVEF* left ventricular ejection fraction, *MI* myocardial infarction, *PCI* percutaneous coronary intervention, *CABG* coronary artery bypass grafting, *ND* not disclosed. Data are mean (standard deviation), median (interquartile range), or number (percentage), as appropriate

### Angiographic characteristics

The summary of the angiographic characteristics of 1406 lesions is provided in Table 3. One of the studies [21] did not provide details of the target vessel. Of the other 9 studies, most CTO lesions involved right coronary artery (RCA, 39%) followed by left anterior descending artery (LAD) at 37.6%. The length of the DCB was 22.7 to 60 millimetres (mm) and the diameter range was from 2.0 to 3.5 mm indicating that target vessels included the whole range of small to large sized coronary vessels. Hybrid strategy involved DES and DCB implantation either as an initial planned strategy (Wang et al. [18]) or as a bailout strategy in cases of flow limiting dissections and threatening abrupt vessel closure (Madanchi et al. [20]) or both (Qin et al. [19]).

**Table 3:** Summary of lesion characteristics of all studies

First author/study/references	LAD *n* (%)	LCx *n* (%)	RCA *n* (%)	J-CTO	DCB length (mm)	DCB diameter (mm)
Scheller et al. [23]	16(47.1)	5(14.7)	13(38.1)	ND	25.60 ± 6.20	2.55 ± 0.42
Onishi et al. [24]	5(42)	1(8)	6(50)	ND	23.75 ± 5.69	2.38 ± 0.2
Basavarajaiah et al. [21]	ND	ND	ND	ND	48.12 ± 25.7	ND
Onishi et al. [25]	5(45)	1(9)	5(45)	ND	22.7 ± 6.1	2.3 ± 0.3
Jun et al. [26]	45(48.4)	24(25.8)	24(25.8)	1.4 ± 0.6	42.3 ± 17.1	2.7 ± 0.4
Zhang et al. [22]	87(41)	26(12)	101(47)	2(1,3)	30(30,60)	3.00(2.50,3.5)
Wang et al. [18]	115(39.7)	59(20.3)	116(40)	1.79 ± 1.07	35.8 ± 19.9	2.63 ± 0.38
Qin et al. [19]	48(31.2)	70(45.5)	36(23.4)	1.5 ± 1.3	30 ± 13.2	2.3 ± 0.3
Terashita et al. [27]	25(30.5)	26(31.7)	31(37.8)	1.7 ± 0.9	47.1 ± 19.7	2.78 ± 0.43
Madanchi et al.[20]	32(29)	22(20)	59(53)	1.8 ± 0.7	ND	2.76 ± 0.51

*DCB* drug-coated balloon, *LAD* left anterior descending, *LCx* left circumflex, *RCA* right coronary artery, *J-CTO* Japanese chronic total occlusion score, *mm* millimetre, *ND* not disclosed. Data are mean (standard deviation), median (interquartile range), or number (percentage), as appropriate

## Systematic review

### Single-arm studies with DCB-only strategy

A total of five single-arm studies is shown in Table 4 as below.

**Table 4:** Summary of DCB-only single arm studies and follow-up (f/u) angiographic outcomes

First author/study/references	Lesion (*n*)	CTO type	RVD, mm at f/u	Late lumen loss, mm at f/u(months)	Binary restenosis *n* (%)	Reocclusion rate *n* (%)
Scheller et al. [23]	34	De novo	2.21 ± 0.58	ND	6(17.6)	2(5.9)
Onishi et al. [24]	12	De novo	2.18 ± 0.53	−0.13 ± 0.61 (7.7 ± 2.8)	2(17)	ND
Onishi et al. [25]	20	De novo	2.49 ± 0.39	−0.45 ± 0.27 (7.2 ± 2.5)	ND	ND
Jun et al. [26]	84	De novo	2.5 ± 0.7	0.03 ± 0.53 (6)	10(14.9)	2(3)
Terashita et al. [27]	82	71denovo and 11 IS-CTO	3.0(2.4–3.2)	−0.15(IQR-0.4to0.23 mm) (8.7 ± 3.9)	12(16.9)	3(4.2)

*DCB* drug-coated balloon, *J-CTO* Japanese chronic total occlusion score, *RVD* reference vessel diameter, *mm* millimetre, *f/u* follow-up, *ND* not disclosed. Data are mean (standard deviation), median (interquartile range), or number (percentage), as appropriate

A feasibility study, conducted by Scheller et al. [23] in 2016, was a multi-center cohort study of 34 patients with de novo CTO recanalized and treated with DCB-only strategy (SeQuent, B. Braun, Germany). Satisfactory recanalization (visual residual stenosis of less than 30% without major dissection) was achieved in 27(79.4%) of patients. Of the 27 patients, restenosis and reocclusion occurred in only 1 patient (3.7%). In the unsatisfactory group of seven patients who were left for evaluation after DCB treatment, three had restenosis and one had reocclusion at follow-up. Significant reduction in Canadian cardiovascular society (CCS) angina class was observed. No death or MI was seen. Late luminal enlargement (LLE) was found in 23 (67.6%) of the patients with a mean late luminal gain of 0.11 ± 0.49 mm at 8.62 ± 9.33 months of follow-up.

Corroborating the above result, Onishi et al. [24] demonstrated a late lumen loss of −0.13 ± 0.61 mm at 7.7 ± 2.8 months post DCB angioplasty in 12 CTO patients in a single-center observational study, restenosis was seen in 2 patients (17%). The same group also showed that LLE following DCB angioplasty occurred more frequently in CTO lesions in their search for predictors of LLE after DCB in de novo coronary artery disease in a retrospective observational study in 2020 [25]. The late lumen loss in the CTO group was −0.45 ± 0.27 mm at 7.2 ± 2.5 months and no TLR was seen in this particular CTO group with LLE at 8 ± 2.7 months of clinical follow-up. Though the vessel size in these three studies was ≤ 2.5 mm, the results clearly demonstrated positive remodeling occurring in small-sized CTO vessels when treated with DCB.

In a retrospective observational study evaluating the long-term clinical outcomes of DCB-only strategy for de novo CTO (*n* = 84), Jun et al. [26] found low rates of hard endpoints and acceptable MACE (composite of CD, non-fatal MI, TVR and TV thrombosis) rates of 8.3% at 1 year and 16.7% at 2 years of follow-up, with a minimal mean late lumen loss of 0.03 ± 0.53 mm at 6 months (n = 61). This study reaffirms the efficacy of DCB in inhibiting negative remodeling in CTO lesions with 55.2% lesions with positive late lumen gain.

Terashita et al. [27] assessed the efficacy of DCB treatment following IVUS guided successful intraplaque wiring and lesion preparation with cutting or scoring balloons in de novo CTO lesions. J-CTO score ≥ 2 was seen in 44 lesions (53.7%) and retrograde procedures were undertaken in 23 (28%) of the 84 lesions. At a median follow-up of 29 months, TLR occurred in 10 (12%) out of 82 lesions. Of the 64 lesions (57 patients) followed up angiographically, 37 (57.8%) exhibited late lumen enlargement and overall, the late lumen loss (LLL) was −0.15 mm (IQR −0.4 to 0.23 mm) at 9 months.

#### DCB vs DES in de novo CTO

Wang et al. [18] conducted a prospective observational study in China, reporting no significant difference in cumulative MACE (composite of all-cause death, TVR and non-fatal MI) at 3 years between DCB strategy (*n* = 290) and DES strategy (*n* = 310) in de novo CTO patients and a significant negative late lumen loss was seen in DCB group (− 0.08 ± 0.65 mm vs 0.35 ± 0.62 mm, *p* < 0.001). The DCB strategy cohort included both DCB only (*n* = 143) and hybrid (DES + DCB, *n* = 147), and their LLL outcomes were reported together. This study demonstrated that DCB can be safely used as an adjunct or definitive treatment for CTO but was a non-randomized observational study.

#### DCB vs DES in de novo and IS-CTO

Madanchi et al. [20] conducted a prospective single-center observational study in a small population of CTO patients from their prospective registries comparing successful CTO-PCI with DCB vs DES. The primary endpoint, MACCE (a composite of CD, TLR, target vessel-MI and stroke) at 12 months, was observed at a rate of 26% in DES group vs 11% in DCB group and cumulative stent length seemed to predict MACCE strongly (HR 1.15 [1.05,1.26] per 10 mm, *p* = 0.003). The DCB group (*n* = 46) included 13 (28%) IS-CTO patients. This is the first prospective study to show a promising better long-term outcome in a DCB-only group with TLR rates of 8% compared to 26% with DES in subgroup analysis and of note, no acute vessel closure was seen in any subgroups.

#### DCB vs DES in IS-CTO

Basavarajaiah et al. [21] performed the first retrospective multi-center observational analysis on long-term outcomes following IS-CTO recanalization with DCB (*n* = 91) vs DES (*n* = 172) vs POBA (*n* = 79). Though the TLR and TVR rates were generally high across three groups, the overall MACE rate (composite of CD, TLR, TV-MI) was numerically lower in DCB group at 34.1% as compared to 44.8% in DES group and 52% in POBA group (*p* = 0.05). An antegrade approach was used in 98.5% of the procedure and 21% of the ISR were in previously placed BMS.

Zhang et al. [22] explored the long-term outcomes of DCB (*n* = 78) vs DES treatment (*n* = 136) for IS-CTO and observed no significant difference in MACE at a median follow-up of 3 years (28.2% in DCB vs 26.5% in DES group) similar to the previous study by Basavarajaiah et al.[21].

#### DCB vs hybrid

Qin et al. [19] conducted a retrospective study looking at clinical outcomes between DCB only (*n* = 97) and hybrid (DES + DCB) group (*n* = 57) in de novo CTO patients. The J-CTO score was higher in hybrid group at 2.0 ± 1.4 compared to DCB-only group at 1.2 ± 1.2. This was associated with greater procedural complexity as evidenced by more frequent retrograde approach, a greater number of CTO wires and a longer procedural time and yet the MACE rate (composite of CD, TVR, TV-MI) was comparable between the groups (13% in DCB vs 12% in hybrid).

## Meta-analysis

Five studies consisting of 1474 patients were included for meta-analysis [18–22].

### Target lesion revascularization (TLR)

**Fig. 2:**
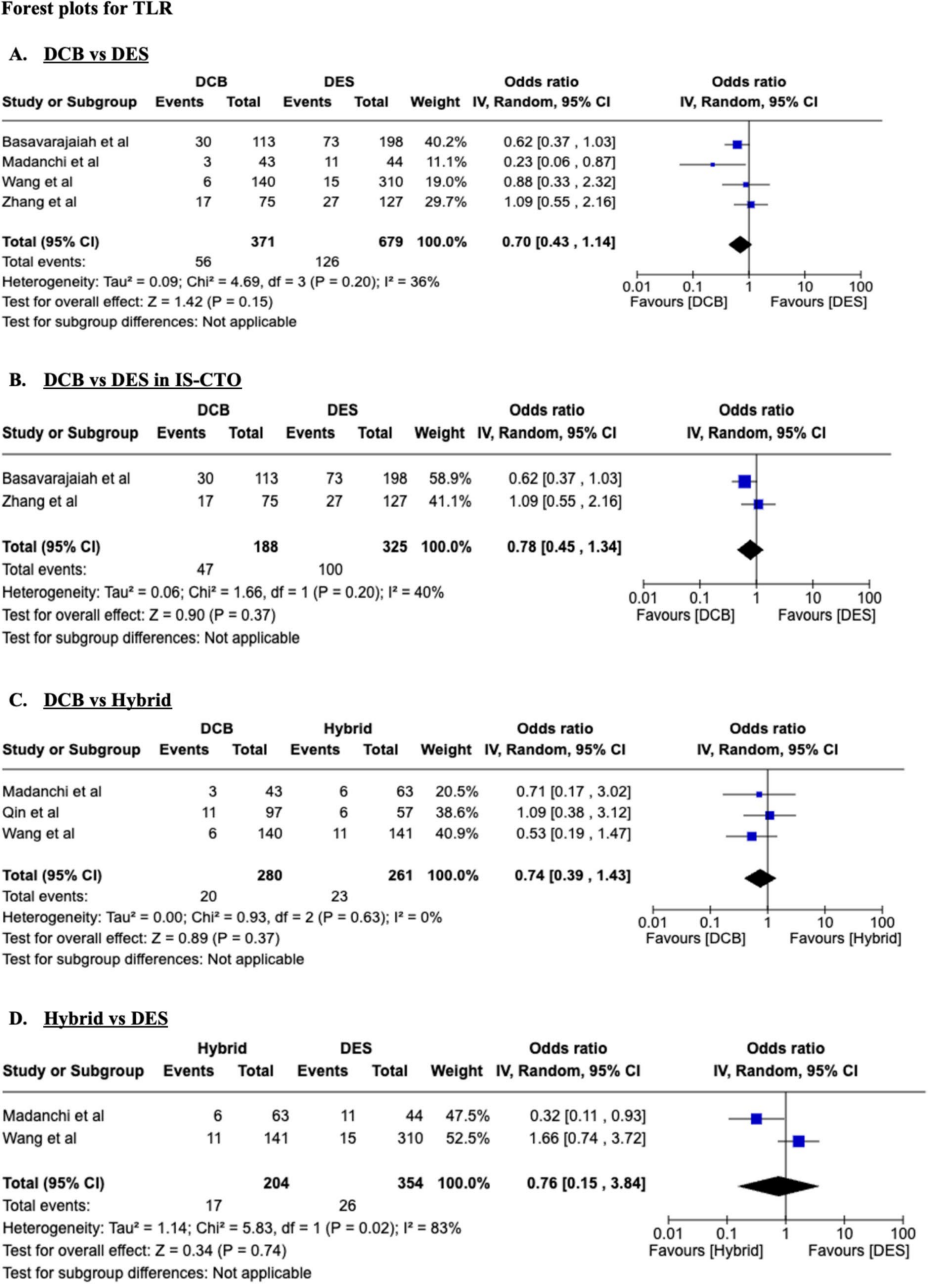
panels A-D: Forest plots for the TLR outcomes in four different groups of comparisons as described. DCB, drug-coated balloon; *DES*, drug-eluting stent, Hybrid = DES + DCB strategy, *CI,* confidence interval; *IV,* inverse-variance pooling method

There were no significant differences in target lesion revascularization in all the four groups, namely DCB vs DES [OR, 0.71; 95% CI 0.49–1.02], DCB vs DES in IS-CTO [OR, 0.78; 95% CI 0.45–1.34], DCB vs hybrid [OR, 0.96; 95% CI 0.39–1.43], and hybrid vs DES [OR, 0.76; 95% CI 0.15–3.84], as shown in Fig. 2.

#### Major adverse cardiac outcomes (composite of TLR, MI, and CD)

**Fig. 3:**
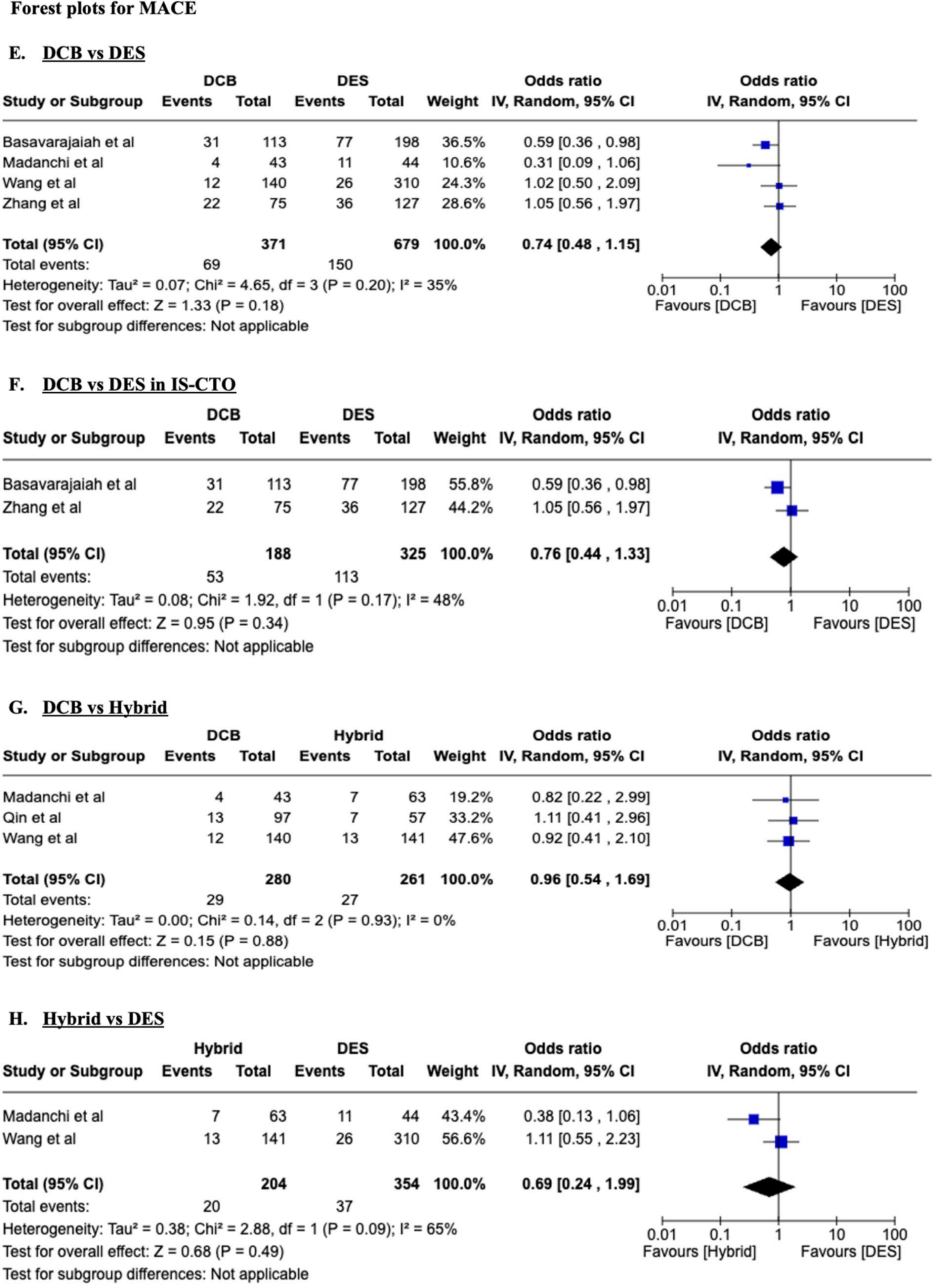
panels E–H: Forest plots for the MACE outcomes in four different groups of comparisons as described. DCB, drug-coated balloon; *DES*, drug-eluting stent, Hybrid = DES + DCB strategy, *CI,* confidence interval; *IV,* inverse-variance pooling method

There were no significant differences in major adverse cardiac events in all the four groups, namely DCB vs DES [OR, 0.74; 95% CI 0.48–1.15], DCB vs DES in IS-CTO [OR, 0.76; 95% CI 0.44–1.33], DCB vs Hybrid [OR, 0.96; 95% CI 0.54–1.69], and Hybrid vs DES [OR, 0.69; 95% CI 0.24–1.99]. A summary of these results is reported in Fig. 3.

#### Cardiac death (CD)

**Fig. 4:**
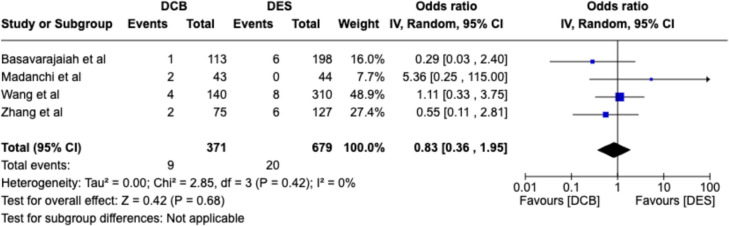
Forest plots for the CD outcomes between DCB vs DES arms in four comparative studies. DCB, drug-coated balloon; *DES*, drug-eluting stent, *CI,* confidence interval; *IV,* inverse-variance pooling method

There was no significant difference in cardiac death after DCB and DES strategies in both de novo and IS-CTO population from four studies as depicted in Fig. 4.

#### Myocardial infarction (any procedural and non-procedural MI)

**Fig. 5:**
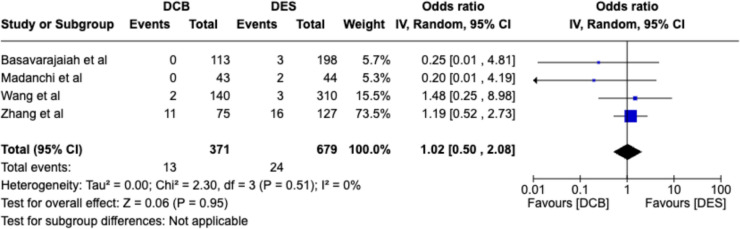
Forest plots for MI between DCB vs DES arms in four comparative studies. DCB, drug-coated balloon; *DES*, drug-eluting stent, *CI,* confidence interval; *IV,* inverse-variance pooling method

An odds ratio of 1.02; 95% CI, 0.50–2.08 was obtained suggesting no significant difference in MI in CTO lesions between DCB and DES strategies. This is illustrated in Fig. 5.

#### Target vessel revascularization (TVR)

**Fig. 6:**
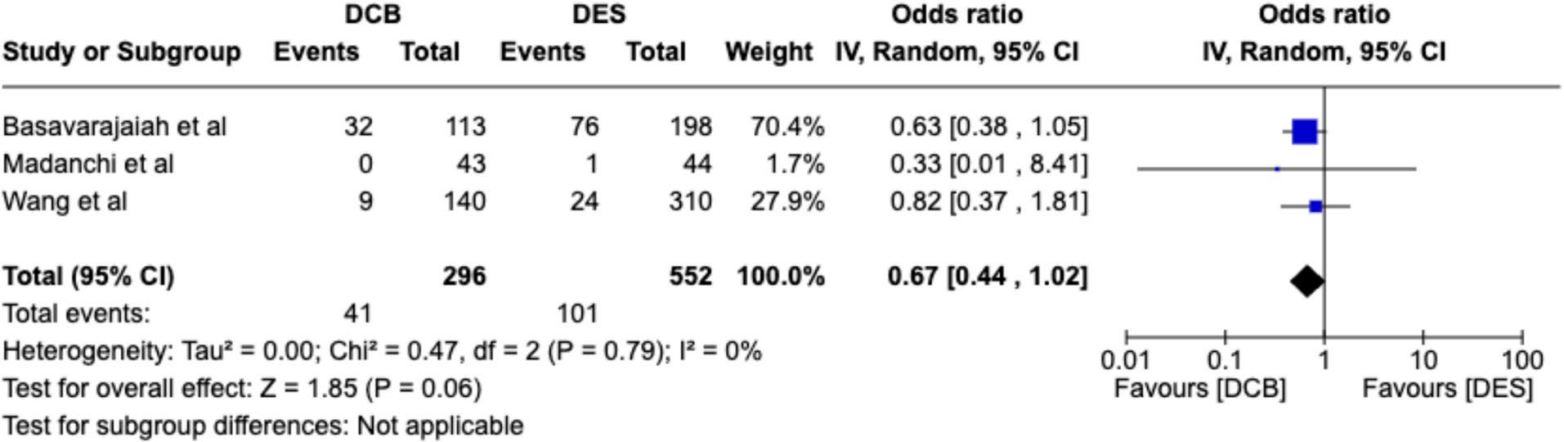
Forest plots for TVR between DCB vs DES arms in CTO studies. DCB, drug-coated balloon; *DES*, drug-eluting stent, IS-CTO, in-stent restenosis, *CI,* confidence interval; *IV,* inverse-variance pooling method

TVR outcomes were available for only three comparative studies and meta-analysis yielded an OR of 0.67; 95% CI [0.44–1.02]. Though there is no statistical significance, the trend seemed to be in favor of DCB in both de novo and IS-CTO population. Figure 6 illustrates these findings.

## Discussion

This is the first systematic review and meta-analysis of DCB CTO studies, including research conducted up to April 2024. The meta-analysis consisted of a total of 1474 patients from 5 comparative studies. The important finding of this study was that there were no significant differences in outcomes (TLR, major adverse cardiac events, CD, MI and TVR) between treatment strategies such as DCB, DES, and hybrid in both de novo and IS-CTO population.

First, this shows DCB-only and hybrid strategies are likely to be a safe and effective therapy in treating both de novo and IS-CTO compared to a DES strategy. Second, TLR rates are comparable between the DCB and DES group. Third, the systematic review of all studies shows that late lumen enlargement is consistently seen across the CTO cohort in 7–12 months following DCB treatment.

One of the most beneficial outcomes of drug-coated balloon in de novo coronary artery disease is late lumen enlargement which occurs frequently at 50–74% during early follow-up phase of intervention [28–30] and the possible mechanisms being either vessel enlargement or regression of plaque or healing of dissection flaps or a combination [31, 32]. Scheller et al. [23] first observed a significant increase in mean lumen diameter from 2.08 ± 0.33 mm to 2.19 ± 0.69 mm at 4–8 months of follow-up post DCB and 67.6% of CTO patients showed late lumen gain due to increased vessel size. In studies conducted by Onishi group [24, 25], LLE occurred frequently in small vessel CTO lesions with moderate length of 16–18 mm that were successfully crossed through true lumen via guidewire and adequately dilated. Jun et al. [26] demonstrated late lumen gain in 55.2% of their patients and minimal late lumen loss (0.03 ± 0.53 mm). Comparing to DES group in a study conducted by Wang et al.[18], LLL was better in DCB group (− 0.08 ± 0.65 mm vs. 0.35 ± 0.62 mm, *p* < 0.001) and it was attributed to enlarged minimum lumen diameter (MLD) in 60.7% of the DCB patients. This phenomenon is crucial particularly in CTO for the following reasons. First, the actual size of the occluded vessel is often unclear angiographically due to extensively disrupted vessel wall architecture, and it is not uncommon to give less attention to stent optimization after a lengthy and onerous procedure leading to under or over expansion [33]. Second, the chronically hypoperfused negatively remodeled small distal vessel of CTO after DES implantation revascularization often undergoes positive luminal gain [34], leading to late acquired stent malapposition. These mechanisms with a stent in situ potentially give rise to late stent thrombosis, in-stent restenosis and target vessel revascularization [35–37]. These can be averted using DCB to deliver the cytostatic drug to freshly opened CTO allowing luminal increase throughout the length of the vessel, thus overcoming the stent-related adverse events.

Furthermore, the rates of TLR and TVR in the DCB-only group are similar to DES group in the above studies. In the recent studies by Jun et al. and Madanchi et al., TLR rates in DCB-only group are 7.1% and 8%, respectively, at 1-year follow-up. At 2-year follow-up, TLR rates were 11% in Jun et al.’s study. Similar rates of TLR and TVR after CTO-PCI with DES are observed in recent registries and RCT [6, 38–41]. In EURO-CTO [41]and PRISON-IV trial [39], 3-year TLR rate of 7%–11.5% was observed in DES CTO group, whereas in J-cypher study, a slightly higher TLR rate of 20.7% was seen at 5 years. TVR is a preferred endpoint to assess patency as per CTO-ARC consortium [1]. TVR rate in Madanchi et al. study was 0% in DCB vs 2.3% in DES group at 1 year, whereas Jun et al. reported an incidence of 11% TVR at 2 years in DCB group. This is comparable with recent CONSISTENT-CTO trial [6] in which TVR rate in DES CTO cohort was 7.1% at 1 year and by 2 years, it increased to 11.9%. In the IS-CTO studies by Basavarajaiah et al. and Zhang et al., TLR rates in DCB group were higher around 33% (42.2% in DES group) and 21.8% (19.9% in DES group), respectively, during long-term follow-up of 4 years. IS-CTO, accounting for 5%–25% of all CTO lesions [42], is generally a very challenging subset to treat percutaneously due to the stent-induced fibrous hyperplasia, multiple layers of overlapping long stents, and higher incidence of balloon undilatable or uncrossable lesions [43]. Although the success rates are now similar to that of de novo CTO-PCI, IS-CTO is associated with higher lesion failure and independently associated with TVR [44]. In a study by Lee et al., DES ISR CTO had significantly worse outcomes of MI [HR: 9.71; 95% CI 2.06–45.81; *p* = 0.004] and TLR[HR: 3.04; 95% CI 1.59–5.81; *p* = 0.001] compared to de novo CTO at 5 years [45]. Multiple stent layers are strong predictors of future repeat revascularization [46] irrespective of the treatment strategy. With these considerations, perhaps PCI in this subset should be undertaken only if it is absolutely indicated as adding more stent layers may increase future failure rates. DCB may, therefore, have a pragmatic benefit by precluding further metal deployment in this challenging IS-CTO population.

While Terashita et al.’s study [27] focused exclusively on lesions recanalized by intraplaque wiring, Qin et al.’s study [19] included two lesions (2.1%) that were recanalized by subintimal tracking subsequently treated with DCB, and five lesions (8.8%) in the hybrid group (DES and DCB). The remaining studies did not provide sufficient technical details to draw any conclusions regarding the outcomes of DCB treatment after successful subintimal tracking and re-entry. There is a concern that DCB application in subintimal recanalization may result in excess enlargement and aneurysm of the vessel wall [47]. Given the abundance of specific binding microtubule in subintimal and adventitial layers, ex vivo studies have shown excess retention and delayed clearance of hydrophobic paclitaxel from these layers [48, 49]. Despite this being a limitation, the novel concept of using DCB after plaque modification (PM) either subintimally or intraplaque or both in failed CTO cases is performed as an investment procedure and is increasingly reported to result in a successful staged procedure [50, 51]. Theoretically, DCB promotes vessel healing in PM-CTO segments and dissection planes enabling distal wiring during staged procedure [52]. IMPROVED CTO (NC05158686) is a multicenter prospective registry investigating this strategy [53].

Finally, DCB is increasingly used as an adjunct in a hybrid approach with DES in resistant acute recoil scenarios and complex procedures involving subintimal tracking and re-entry where a metallic scaffold is needed to maintain patency and adequate distal perfusion. These outcomes are no different to DES-only strategy according to our study.

In a meta-analysis of 17 studies comparing PCI and medical therapy for CTO, Li et al. [9] demonstrated a higher risk of all-cause mortality, cardiac death, and MI with medical therapy compared to PCI strategy with DES. Our work showed CTO-PCI with DCB has no significant differences in similar outcomes when compared to DES strategy. It is reasonable to assume that patients with CTO and reversible ischemia could benefit from revascularization using a DCB strategy compared to medical therapy. However, a randomized controlled trial is necessary to confirm this assumption.

## Limitations

Our study has few limitations. First, there are only a few studies in the field with relatively small numbers of patients included. Second, since there are no available randomized controlled trials (RCTs), our study has only included observational studies. Third, there is significant heterogeneity in study methodology and statistical heterogeneity. To address these issues, we conducted several subgroup meta-analyses and used a random effects model to account for the statistical heterogeneity. Current guidelines recommend CTO-PCI primarily for symptom benefit, and this clinical outcome was not measured in any of the studies except one. Larger studies with adequate power and consensus-based uniform safety endpoints are needed to compare each distinct treatment strategy (DCB only, DES only, hybrid) in both de novo and IS-CTO groups individually.

## Conclusion

Current evidence suggests that DCB may be a safe and effective alternative or an adjunct to DES in treating coronary CTO, including de novo and IS-CTO lesions. There is a consistent pattern of late lumen gain in CTO lesions after DCB angioplasty, and acceptable rates of hard end points are observed.

## Supplementary Information

Below is the link to the electronic supplementary material.

Supplementary file 1 (DOCX 32 kb)

## Data Availability

All data supporting the finding of this study are available within the paper and its supplementary information.
